# The First Pan-American Medical Congress

**Published:** 1893-10

**Authors:** 


BUFFALO MEDICAL AND SURGICAL JOURNAL

A MONTHLY REVIEW OF MEDICINE AND SURGERY.

*EDITORS:*

THOMAS LOTHROP, M. D. - - WM. WARREN POTTER, M. D.

All communications, whether of a literary or business character, should be addressed to the managing editor: 284 FRANKLIN STREET, BUFFALO, N. Y.

VOL. XXXIII. OCTOBER, 1893. No. 3.

### THE FIRST PAN-AMERICAN MEDICAL CONGRESS, WASHINGTON, D. C., 1893.

THE First Congress of the medical profession of all the Americas, has met, discussed the questions for which it was convened, and adjourned. Its record has passed into history, and will furnish many a theme for reflection during many years to come. There are some points which it seems within the province of the journalist to comment upon, even though it is yet too soon to form a complete judgment as to the far-reaching benefits that will accrue from this remarkable meeting.

In the first place, it must be understood that this Congress was an independent organization, with its own autonomy from start to finish. While it is true that the Organization Committee was made up by appointment of the American Medical Association, yet, as soon as the Congress became incorporated, it ceased to have any relationship to the American Medical Association, or to owe any fealty to that body. It seems necessary to make this clear at the outset, for the reason that it has been asserted that some prominent physicians remained away from the Congress because it was supposed to be a creature of the American Medical Association. If any such there be, let them be pitied; we fear they made a serious mistake, besides having shown, by their conduct, on what small foundation their mentality is constructed.

The Pan-American Medical Congress possesses the power of self-perpetuation, and has already appointed the second Congress to convene in the City of Mexico, three years hence.

The opening address, by the President of the United States, was in singularly good taste, not only with reference to its material, but also with regard to its length. Brief, concise, finished, it is, taken altogether, one of the best public addresses that His Excellency has made.

The President of the Congress, Dr. William Pepper, of Philadelphia, was an ideal presiding officer. His practical knowledge and tact, together with his wit, wisdom, and acumen, equipped him with all that was needful for the occasion, and it is hardly necessary to add that the best possible use was made of all this varied and diversified talent. Dr. Pepper's address was a scholarly paper, that will take rank among the first of such, and should be read by every thoughtful physician in the Western hemisphere.

The attendance upon the Congress was very large, even more so than could reasonably have been anticipated, for the enormous number of 1,500 was borne upon the register as early as the second day of the meeting. The total registration was several hundred greater. The foreign delegates were there in goodly numbers, and were representative men in every sense of the word.

The work of the sections was judged by competent observers to be above the average at such meetings. Several of the sections were very largely attended, and in them discussions were both animated and instructive to an extreme degree. It is fair to say that the congress was subdivided into too many sections to make its work the most effective, and its experience will serve to benefit the organization of future congresses. The sections on medicine, surgery, obstetrics, gynecology, pedagogy, and hygiene, were especially interesting, and the debates were participated in by representative men from every portion of the Western hemisphere.

It is to be regretted that it became necessary to fix the date of the meeting so early in the season, on account of the hot weather which nearly always prevails in Washington during September. It was originally thought to name the first Tuesday of October as the date of meeting, and this would have been much the better time. But it was ascertained that that date would conflict with the Eleventh International Medical Congress at Rome. Since it has been necessary to postpone the latter, we regret that October was not chosen instead of September for the congress.

The founder of the congress, Dr. Charles A. L. Reed, of Cincinnati, can ever look back upon his work, arduous though it has been for more than two years, with the satisfaction that he has been supported by the best men in the profession that

America can produce, supported loyally and faithfully from start to finish, and he will always be able to point with great pride to the fact that the First Pan-American Medical Congress was the creature of his own brain, and its great success will ever be a monument to his skill, erudition, and executive talent. All praise, then, say we to the father and secretary-general of the First Pan-American Medical Congress. The Congress was not merely a success—it was a triumph.

